# Characterization and validation of electronic medical record data for pharmacoepidemiologic research

**DOI:** 10.3389/fphar.2026.1812502

**Published:** 2026-07-21

**Authors:** Tyler Schneider, Tanvi Punjani, Jessyca Matos Silva, Lawrence Mbuagbaw, Paul Gibson, Anne M. Holbrook

**Affiliations:** 1 Clinical Pharmacology Research, Research Institute of St Joe’s Hamilton, Hamilton, ON, Canada; 2 Department of Pharmacy, Hamilton Health Sciences, Hamilton, ON, Canada; 3 Department of Health Research Methods, Evidence and Impact (HEI), McMaster University, Hamilton, ON, Canada; 4 Department of Anesthesia, McMaster University, Hamilton, ON, Canada; 5 Department of Pediatrics, McMaster University, Hamilton, ON, Canada; 6 Biostatistics Unit, Research Institute of St. Joe’s Hamilton, Hamilton, ON, Canada; 7 Centre for Development of Best Practices in Health (CDBPH), Yaoundé Central Hospital, Yaoundé, Cameroon; 8 Division of Epidemiology and Biostatistics, Department of Global Health, Stellenbosch University, Cape Town, South Africa; 9 Division of Haematology/Oncology, McMaster Children’s Hospital, Hamilton, ON, Canada; 10 Division of Clinical Pharmacology and Toxicology, Department of Medicine, McMaster University, Hamilton, ON, Canada

**Keywords:** data accuracy, data validation, electronic medical records, epic, pharmacoepidemiology

## Abstract

**Purpose:**

Electronic medical records (EMRs) are essential for pharmacoepidemiologic research; however, information on the quality of Canadian hospital EMR data is scarce. We aimed to test data validity in the Epic EMR for key pharmacoepidemiologic themes using QT-prolonging medications and major adverse cardiac events (MACEs) as the research question.

**Methods:**

An entity relationship diagram (ERD) was developed to navigate >20,000 tables. Computational data validation (comparison with Epic SlicerDicer and Canadian Institute for Health Information Discharge Abstract Database (CIHI-DAD) data) and manual data validation (chart review) were applied with iterative adjustments. Percent agreement with 95% confidence intervals was used to estimate data validity.

**Results:**

The ERD demonstrated that 43 tables are needed for key pharmacoepidemiologic research data. A cohort of 70,078 adult inpatients was used for computational validation and 2,281 patient charts for manual validation. Demographics (for example, age, sex, gender, admitting service, and critical care transfers); exposures (medication administrations); many potential confounders (lab results, interacting drugs, and electrocardiograms [EKGs]); and most timestamping (admission, medication administrations, death, lab results, and EKGs) were validated with >95% agreement. Inadequate agreement for MACE (other than death) brought the primary outcome MACE agreement to <90%, requiring patient-level linkage to CIHI-DAD to improve agreement. Comorbidities, including hypertension and heart failure, also required this linkage.

**Conclusion:**

A world-leading proprietary EMR after the painstaking development of an ERD and validation of key data fields showed that not all data are of sufficient quality for pharmacoepidemiologic research. Linked human-coded data are required for some diagnoses.

## Key points

- Electronic medical records are rich in patient data that must be validated for research use.
- Epic does not provide an entity relationship diagram identifying data field locations or linkages.
- A few available data tables are required for pharmacoepidemiologic research.
- Most key data concepts had >95% agreement with manual chart review or computational validation.
- Accurately identifying diagnoses and their timing requires linked data or natural language processing.

## Introduction

Electronic medical records (EMRs) capture an abundance of data from patients while helping with clinical workflows and administrative processes and are also very useful for research to improve the quality of care (Ehrenstein et al., 2019). However, clinical data may not necessarily be of adequate quality for research. Making actionable conclusions that will alter clinical flow or health policies relies on data of low bias, which requires ensuring the relevance, accuracy, and completeness of the data used (Ehrenstein et al., 2019; Wang et al., 2023).

Epic is a leading EMR worldwide and is the most common EMR in the United States and Canada, despite only being first implemented in Canada in 2017 (Dyrda, 2024; Witowski, 2025; Guran, 2017). The Epic EMR has been used for many types of research, including clinical, health service, population health, quality improvement, registry, and economic analyses (Chishtie et al., 2023). For pharmacoepidemiologic research, EMR data have been linked to other data sets such as pharmacy records, claims data, and clinical research networks (Wu et al., 2025; Geddes et al., 2025; Beukelman et al., 2023).

Previous studies have validated parts of an EMR but not all required themes for pharmacoepidemiologic research. EMR data from two hospitals in the Netherlands were validated, showing ≥98% agreement for patient demographics and blood products used (Hoeven et al., 2017). An EMR data validation study in Spain was computationally validated against a research database that reported positive predictive values (PPVs) for 12 sections of the EMR, including demographics, comorbidities, medications, and microbiology, which ranged from 58% to 99% (Pedrera-Jimenez et al., 2022). The Hospital for Sick Children developed a research database with 18 curated tables for Epic, which was validated iteratively using manual chart review until all errors were corrected (Guo et al., 2023). However, the validation does not describe how diagnoses or medication administrations were validated (Guo et al., 2023).

**FIGURE 1:**
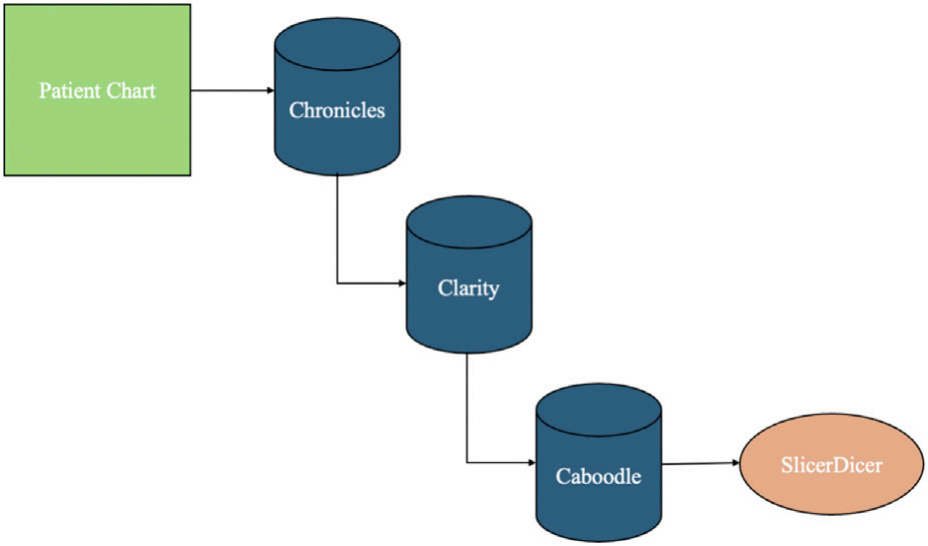
Epic data flow and sources data flow and sources for the epic electronic medical record adapted from the Clinical and Translational Science Institute (2025). Patient chart: the medical record for an individual patient in epic. Chronicles: the main database of Epic; non-relational database (Guo et al., 2023; Newman, 2024). Clarity: relational database with data from Chronicles that can be used for detailed and advanced data reports (Guo et al., 2023; Newman, 2024). Caboodle: data warehouse containing an abundance of data from clarity which can display reports with epic and non-epic data that has been incorporated (Guo et al., 2023; Newman, 2024). SlicerDicer: epic tool displaying de-identified high-level patient data based on Caboodle (Saini et al., 2021).

Conducting validation studies is not straightforward. Most proprietary EMR systems, including Epic, provide no clear entity relationship diagram (ERD) outlining where data reside, table organization, or relationships. Epic contains three databases: Chronicles, Clarity, and Caboodle (Figure 1) (Guo et al., 2023; Clinical and Translational Science Institute, 2025; Newman, 2024). Chronicles is the real-time clinical database, Clarity is used for large and complex data reports and is most suitable for research, and Caboodle is used to produce reports and supply data for tools such as SlicerDicer, which provides high-level, deidentified data on selected patient populations (Guo et al., 2023; Newman, 2024; Saini et al., 2021). Identifying relevant data fields, tables, and their linkages is challenging because the Epic Clarity database contains thousands of data tables (Guo et al., 2023; PennDnA, 2025). Before using EMR data for research, we must be assured that the data are relevant, accurate, and complete. This requires an understanding of key concepts and requirements for data validation, as well as the ability to internally and externally validate data and iteratively correct errors (Newton et al., 2013).

As part of a research program to clarify the association between “known” QT-prolonging (QTP) medications (QTPmeds) and relevant major adverse cardiac events (MACEs) using the Epic EMR at our institution, we required the validation of key pharmacoepidemiologic variables in our EMR. This research could lead to streamlining the plethora of medication-related safety alerts for QTPmeds, many of which are based on theoretical or very low-quality data (Berger et al., 2021; Nham et al., 2024; Garcia et al., 2024).

## Methods

### Study design

This study is reported on the basis of the recommendations of the STAtement on the Reporting of Evaluation studies in Health Informatics (Supplementary Material Table A.1) (Brender et al., 2013). This is a retrospective validation study using Epic (institutionally known as Dovetale) EMR data of all adult inpatients aged ≥18 years admitted to our hospital from 2 December 2017 to 24 March 2023. The Hamilton Integrated Research Ethics Board provided a waiver of consent for this study due to its retrospective nature and large cohort. Our hospital is in Canada and is composed of three sites: an acute care hospital with over 600 beds, which includes a women’s and infant’s department, an inpatient mental health hospital, and an outpatient urgent care and services center (St. Joseph’s Healthcare Hamilton, 2025).

### Methods for data acquisition and measurement

**FIGURE 2:**
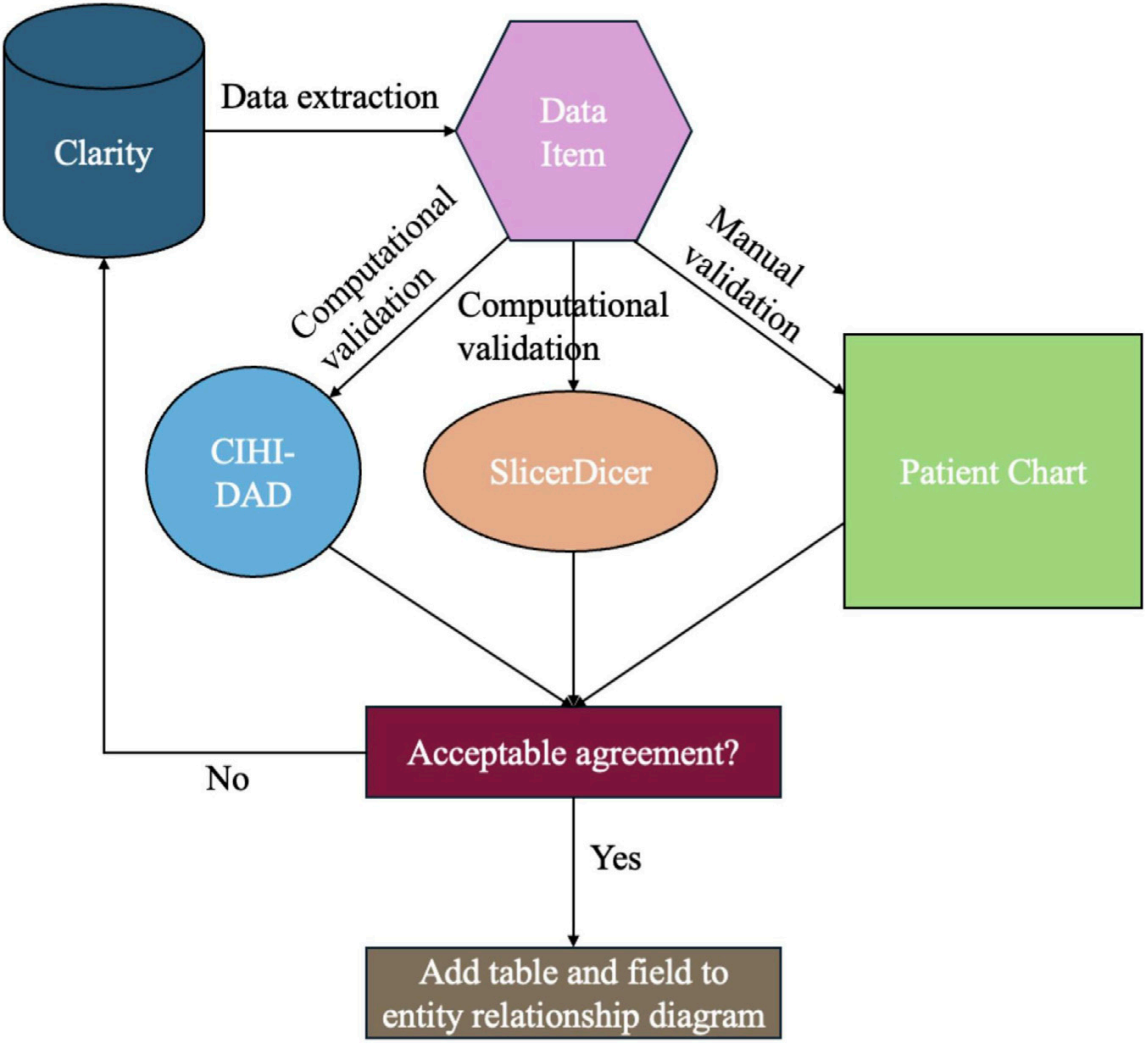
Data extraction and validation process. CIHI-DAD, Canadian institute for health information discharge abstract database.

Demographic and clinical data for eligible patients were extracted from the Epic Clarity database by an Epic-certified senior research data analyst using structured query language (SQL) queries (see Figure 2 for full data workflow). Data tables and fields in the Epic Clarity database were identified using the Clarity Dictionary in the Epic Data Handbook (Epic Systems, 2025a). The data were manually and computationally validated (if data were available for comparison). The SQL queries were iteratively adjusted to improve the completeness and correctness of the data extractions by changing the data tables, fields, and filters. Epic-certified clinical informatics specialists at our institution were consulted when fields were missing data. When the accurate data tables and fields were located, they were added to the ERD.

Five key data themes required for pharmacoepidemiologic research were selected for validation: demographics, exposures, outcomes, potential confounders, and timestamping. In this study, MACEs were our primary outcome and followed the Food and Drug Administration QTP standard outcomes but adapted for the additional data available in the hospital: death, ventricular tachycardia including Torsades de Pointes (TdP), ventricular fibrillation, nonfatal cardiac arrest, syncope, and seizure (Food and Drug Administration, 2005). These outcomes were extracted using the International Classification of Diseases, 10th Revision (ICD-10) codes or diagnosis names (Supplementary Material Table A.2). Comorbidities were also extracted using ICD-10 codes or diagnosis names. The ability to identify MACE occurring after admission was essential because research requires exposures to predate outcomes. For validation, outcomes were considered to have happened if they were documented in a chart note as being witnessed by a healthcare professional, documented in a physician note, or confirmed with testing.

Manual data validation was performed as the gold standard for completeness and correctness of each item by extracting data from the Epic Clarity database and comparing it with data found through random patient chart review from the research view of Epic (Verma et al., 2021). Search strings and Boolean operators were used for chart review, including comorbidities and lab results (Supplementary Material Table A.3). Computational data validation was also performed when data were available by comparing Epic extractions against the Canadian Institute for Health Information Discharge Abstract Database (CIHI-DAD) to check for completeness and correctness, or Epic was computationally validated against itself using similar items or the Epic tool SlicerDicer for internal concordance.

CIHI-DAD is the reference database for demographic, clinical, and administrative data for hospital inpatient discharges from facilities across Canada, except Quebec (Canadian Institute for Health Information, 2015). CIHI-DAD data are recorded by professional coders who review patient charts and are used by leading health services research organizations in Canada (Canadian Institute for Health Information, 2016). The agreement in CIHI-DAD for demographics and hospital admission information is very high (>90%) (Canadian Institute for Health Information, 2016). The agreement for diagnoses was validated in CIHI-DAD against the chart with agreement of 77%–80%, and agreement for diagnoses in the chart present in CIHI-DAD was 83%–84% (Canadian Institute for Health Information, 2016). CIHI-DAD was used to validate age, hospital admission including the admission date, MACE including death, and comorbidities. SlicerDicer was used to internally check the completeness of our Epic Clarity extractions, specifically the number of eligible patients, age, and sex distribution. SlicerDicer provides total numbers, percentages, and minimums and maximums based on the selected criteria (UC Davis Health, 2025; Ford et al., 2018).

We followed the recommended eight methods to assess the dimensions of data quality: element presence by ensuring expected and desired data concepts are available; data element agreement by comparing items within the EMR for similar information; distribution comparison using SlicerDicer for aggregate statistics; data source agreement by comparing EMR data with another source (SlicerDicer) for agreement; comparing EMR data with a gold standard such as CIHI-DAD data; log review using timestamping to assist with validation; validity checks by looking at extremes of age- and sex-based procedures; and structural agreement by looking at lab units and diagnostic codes for formatting agreement (Lewis et al., 2023; Weiskopf and Weng, 2013).

### Methods for data analysis

Data analysis was conducted using Microsoft® Excel. Descriptive statistics (mean, median, standard deviation, and interquartile range) were used to characterize the cohort. Percent agreement reported with 95% confidence intervals (CIs) calculated with WINPEPI was used for all items validated by comparing the data extraction with the chart or comparator data set (Abramson, 2011). Diagnostic accuracy measures of sensitivity, specificity, PPV, and negative predictive value (NPV) were also used for medication exposures and outcomes (Shreffler and Huecker, 2023). Each data concept was manually and computationally validated if data for comparison were available. Percent agreement was considered excellent if it was >95% and inadequate if it was <80%.

## Results

### Entity relationship diagram

**FIGURE 3:**
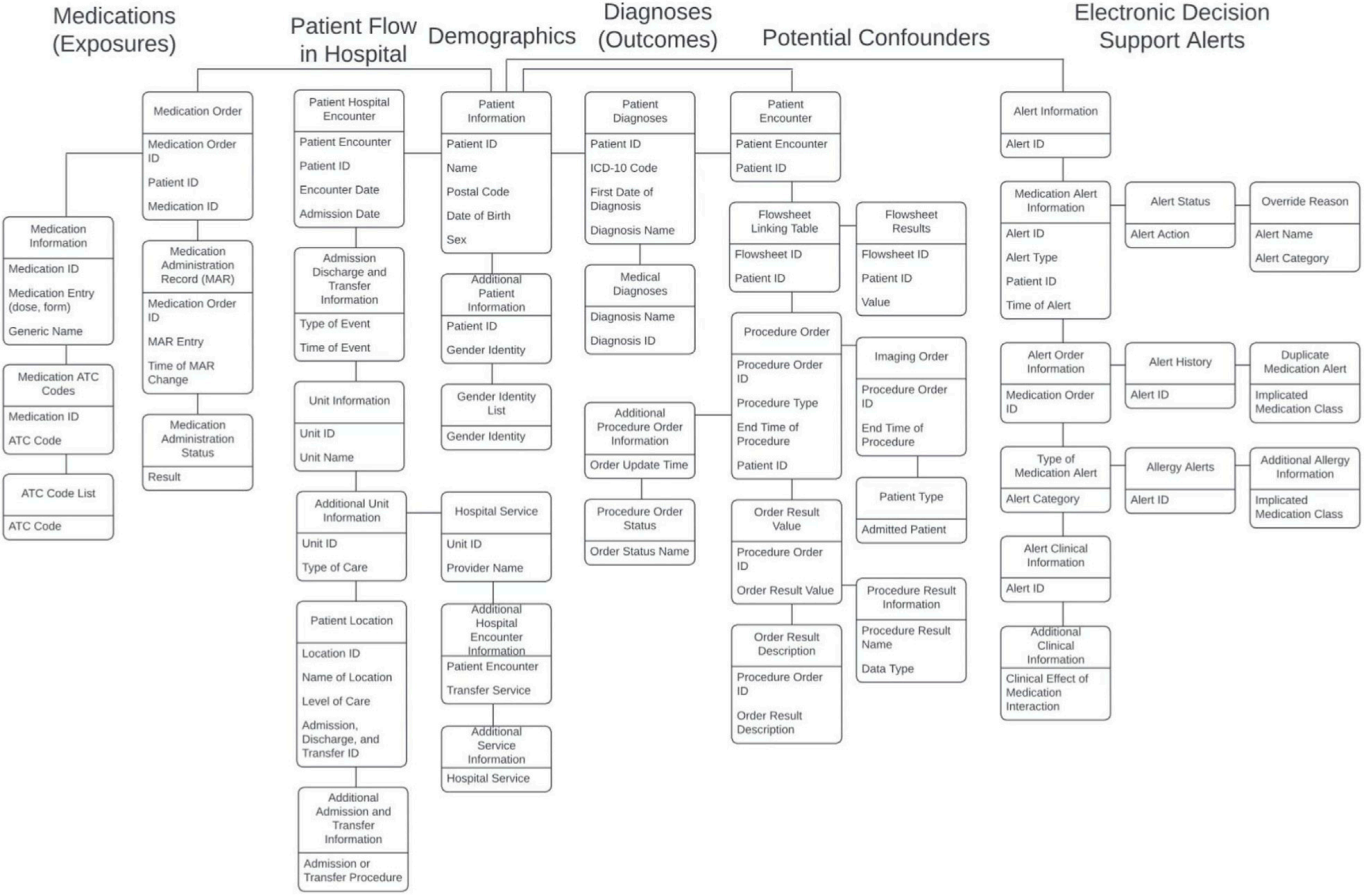
OMOP style entity relationship diagram for Epic data (Schuemie and Los, 2025). ATC, Anatomical therapeutic chemical; MAR, medication administration record; ICD-10, International classification of diseases, 10th revision.

An ERD was developed and converted to the style of the Observational Medical Outcomes Partnership (OMOP) common data model showing example linkages between relevant data tables (Figure 3) (Schuemie and Los, 2025). The Epic Clarity database at our institution included 20,764 tables (L. Bishop, email, 17 April 2024). Iterative searches for relevant research data eventually identified 43 tables containing the data required for this project.

### Study cohort and data validation

The characteristics of the study cohort are shown in Table 1. The details of data validation are shown in Table 2.

**TABLE 1:** Characteristics of the study cohort.

Characteristic	Number (%)
Patients	70,078
Hospital admissions	108,598
Hospital admissions per patient - Mean - Median	1.55 (SD^a^ 1.5) 1 (IQR^b^ 1)
Age at admission (mean [SD]; median [IQR]) - Mean - Median	55.5 (SD 20.8) 58 (IQR 37)
Sex (admissions) - Female - Male	64,105 (59.0%) 44,493 (41.0%)
Gender identity (admissions) - Female - Male - Non-binary - Other - Missing data	3,409 (3.1%) 3,517 (3.2%) 218 (0.2%) 118 (0.1%) 101,336 (93.3%)
Admitting unit - General internal medicine - Nephrology - General surgery - Orthopedics - Urology - Thoracic surgery - Head and neck - Obstetrics - Gynecology - Acute mental health	25,021 (23.0%) 4,728 (4.4%) 14,113 (13.0%) 6,863 (6.3%) 6,649 (6.1%) 4,425 (4.1%) 2,715 (2.5%) 16,951 (15.6%) 2,169 (2.0%) 7,125 (6.6%)
Patients transferred to critical care	12,138 (17.3%)
Patients exposed to at least 1 “known” QTPmed^c^	50,487 (72.0%)
Patients not exposed to any “known” QTPmeds	19,591 (28.0%)
Patients not exposed to any QTPmeds	5,174 (7.4%)
Patients who experienced at least 1 MACE^d^ outcome - Nonfatal MACE - Death - Total	Nonfatal MACE: 5,369 Death: 3,775 Total: 9,144 unique patients (13.0%)
EKGs^e^ - Patients - Number of EKGs	32,767 (46.8%) 98,814
Lab tests – potassium - Patients - Number of lab tests	Patients: 54,467 (77.7%) Number of lab tests: 557,577 (54.9%)
Lab tests – calcium - Patients - Number of lab tests	30,515 (43.5%) 169,839 (16.7%)
Lab tests – magnesium - Patients - Number of lab tests	30,404 (43.4%) 180,858 (17.8%)
Lab tests – troponin - Patients - Number of lab tests	28,401 (40.5%) 107,141 (10.6%)
Telemetry - Patients - Number of telemetry orders	10,467 (14.9%) 17,232

^a^SD, standard deviation.

^b^IQR, interquartile range.

^c^QTPmed, QT-prolonging medication.

^d^MACE, major adverse cardiac events.

^e^EKG, electrocardiogram.

**TABLE 2:** Data validation items and results for Epic.

Item to be validated	Validation standard (SlicerDicer, CIHI)	Number of charts checked	Results (% agreement)	95% confidence interval
*1. Demographics*				
Total number of patients in cohorts	SD^a^	70,078	>99%	(99.8, 99.9)
Patients with >1 MRN	N/A	70,078	100%	(100, 100)
Correct age >18 years on admission date	DAD^b^	10,065	>99%	(99.3, 99.6)
Age <18 years	DAD	10,065	100%	(100, 100)
Date of birth	MV	200	100%	(98.2, 100)
Implausible age	MV, SD	13	100%	(75.3, 100)
Hospital encounters (potential missed admissions)	MV^c^	300	100%	(98.8, 100)
Sex	MV, SD	131	100%, 99%	(97.2, 100), (99.8, 99.9)
Gender	MV	131	100%	(97.2, 100)
Implausible sex-based procedures (e.g., males delivering babies and females having prostate surgery)	MV	4	>99%	(99.9, 100)
Postal code	MV	200	100%	(98.2, 100)
Admission	MV, DAD	50, 10,065	100%, 99%	(92.9, 100), (99.7, 99.9)
Admitting service	MV	50	100%	(92.9, 100)
Presence in critical care	MV	79	100%	(95.4, 100)
*2. Exposures*				
Patients given “known” QTPmeds	MV	235	97%	(94.0, 98.8)
Patients not given any “known” QTPmeds	MV	100	99%	(94.6, 99.9)
Patients not given any QTPmeds	MV	100	96%	(90.0, 98.9)
*3. Outcomes*				
Patients with at least 1 MACE outcome (ventricular arrhythmia composite, cardiac arrest, syncope, seizure, and death)	MV	250	70%	(63.9, 75.6)
Death	MV, DAD	200,444	100%, 100%	(98.2, 100), (99.2, 100)
Ventricular arrhythmia composite (any of ventricular tachycardia including TdP^d^ and ventricular fibrillation)	MV	50	42%	(28.2, 56.8)
Cardiac arrest	MV	50	84%	(70.9, 92.8)
Syncope	MV	50	66%	(51.2, 78.8)
Seizure	MV	50	58%	(43.2, 71.8)
Patients with no MACE outcome	MV	100	98%	(93.0, 99.8)
*4. Potential confounders*				
Lab values (potassium, magnesium, calcium, and troponin)	MV	378 lab results for 50 patients	100%	(99.0, 100)
EKG^e^ data (for QTc-B^f^ and cardiac rhythm interpretation)	MV	117 EKGs for 63 patients	99%	(95.3, 99.9)
Telemetry order	MV	87 orders for 50 patients	95%	(88.6, 98.7)
Telemetry indication	MV	84 orders for 50 patients	98%	(91.7, 99.7)
Diabetes	MV, DAD	75,560	100%, 56%	(95.2, 100), (51.9, 60.2)
Hypertension	MV, DAD	75,502	100%, 84%	(95.2, 100) (80.4, 87.0)
Heart failure	MV, DAD	75,167	96%, 69%	(88.8, 99.2), (61.9, 76.3)
158, 210, and 172 administrations for 6, 25, and 25 patients, respectively	MV	158, 210, and 172 administrations for 6, 25, and 25 patients	100%	(99.3, 100)
Metabolic inducer (phenytoin)	MV	199 administrations for 25 patients	100%	(98.2, 100)
Diuretics (furosemide, hydrochlorothiazide, indapamide, and metolazone)	MV	384 administrations for 25 patients	93%	(90.5, 95.7)
Electronic decision support alerts shown to providers	MV	65 alerts for 25 patients	88%	(77.2, 94.5)
Alerts being shown to the listed provider	MV	57 alerts	100%	(93.7, 100)
Alerts being overridden	MV	57 alerts	100%	(93.7, 100)
*5. Timestamping*				
Date of admission	DAD	10,065	99%	(98.7, 99.1)
Minute of medication administration	MV	56 patients	100%	(93.6, 100)
Date of nonfatal MACE	MV	25 patients	36%	(18.0, 57.5)
Date of death	MV	200	98%	(94.3, 99.2)
Minute of lab draw	MV	378 lab results	100%	(99.0, 100)
Minute of EKG	MV	67 EKGs	100%	(94.6, 100)
Minute of telemetry order	MV	84 orders	100%	(95.7, 100)
Minute of telemetry discontinuation	MV	84 orders	87%	(77.8, 93.3)
Minute of alerts	MV	57 alerts	100%	(93.7, 100)

^a^SD, SlicerDicer.

^b^CIHI-DAD, Canadian Institute for Health Information Discharge Abstract Database data.

^c^MV, manual validation.

^d^TdP, Torsades de Pointes.

^e^EKG, electrocardiogram.

^f^QTc-B, QT-interval corrected using Bazett’s formula.

### Study findings and outcome data

#### Demographics

The total number of patients in the cohort was 70,078; this was computationally validated against SlicerDicer, which returned 70,170 patients with >99% agreement (95% CI: 99.8, 99.9). Patients with >1 medical record number (MRN) were validated by checking for health card numbers associated with multiple MRNs. A sample of patients manually validated for >1 MRN, age at the time of admission and implausible age, date of birth, hospital admission, sex and implausible sex, postal code, presence in critical care, and provider service showed 100% agreement. Gender identity was manually validated for 131 charts showing 100% agreement; however, it was only available for 7,262 (6.7%) admissions. Sex was computationally validated against SlicerDicer, in which both Epic and SlicerDicer resulted in 60.8% and 39.2% female and male patients, respectively. Admission and age were computationally validated against CIHI-DAD with >99% agreement. Overall, all demographic items had excellent validity (>95%).

#### Exposures

Exposures included administration of QTPmeds. The “known,” “possible,” and “conditional” lists of QTPmeds were extracted from CredibleMeds (CredibleMeds, 2024). The drug name and administration of “known” QTPmeds were manually validated for 235 charts with 97% (95% CI: 94.0, 98.8) agreement. Patients not given any “known” QTPmeds and no QTPmeds of any type were manually validated for 100 charts with 99% (95% CI: 94.6, 99.9) and 96% (95% CI: 90.0, 98.9) agreement, respectively. Diagnostic accuracy for exposure to “known” QTPmeds based on manual validation of 335 charts showed a sensitivity of 99% (95% CI: 97.6, 99.9), a specificity of 93% (95% CI: 86.9, 97.3), a PPV of 97% (95% CI: 94.1, 98.5), and an NPV of 99% (95% CI: 93.3, 99.9) (Table 3). Some medication administrations were missed because of limitations when combining Anatomical Therapeutic Chemical (ATC) code and generic name data extractions, where certain dosage forms such as half tablets could not be extracted. Overall, exposures to QTPmeds had excellent validity (>95%).

**TABLE 3:** Diagnostic accuracy measures for Epic exposures to “known” QTPmeds.

True positive: 228	False positive: 7	PPV^a^: 97% (95% CI: 94.1, 98.5)
False negative: 1	True negative: 99	NPV^b^: 99% (95% CI: 93.3, 99.9)
Sn^c^: 99% (95% CI: 97.6, 99.9)	Sp^d^: 93% (95% CI: 86.9, 97.3)	n = 335 patients

^a^PPV, positive predictive value.

^b^NPV, negative predictive value.

^c^Sn, sensitivity.

^d^Sp, specificity.

#### Outcomes

Our MACE outcomes of interest included death, ventricular arrhythmia composite (any ventricular tachycardia including TdP and ventricular fibrillation), nonfatal cardiac arrest, syncope, and seizure. MACEs were manually and computationally validated, demonstrating 100% agreement for death. Epic does not differentiate between the phase of care when storing diagnoses in structured data fields; thus, manual validation was conducted on the basis of whether the outcome occurred while the patient was admitted or as a presenting diagnosis rather than in the past. Agreement for the ventricular arrhythmia composite was 42% (95% CI: 28.2, 56.8), nonfatal cardiac arrest was 84% (95% CI: 70.9, 92.8), syncope was 66% (95% CI: 51.2, 78.8), and seizure was 58% (95% CI: 43.2, 71.8). The diagnostic accuracy of MACE outcomes based on manual validation of 250 patients with MACEs and 100 patients without MACEs showed a sensitivity of 99% (95% CI: 96.0, 99.9), a specificity of 57% (95% CI: 48.9, 64.2), a PPV of 70% (95% CI: 66.3, 73.5), and an NPV of 98% (95% CI: 92.5, 99.5) (Table 4). This indicated an unacceptably high rate of false-positive diagnoses in Epic due to two main problems with the EMR data: (a) diagnostic uncertainty (e.g., unwitnessed events like syncope or seizure) and (b) Epic not differentiating the phase of care as some diagnoses were historical but remain on the chart. By adding patient-level linkage of Epic data to CIHI-DAD, which differentiates the phase of care, manual validation for 300 patients identified as having MACE outcomes after admission (other than death) improved to 79% (95% CI: 74.3, 83.8) agreement.

**TABLE 4:** Diagnostic accuracy measures for Epic MACE outcomes.

True positive: 175	False positive: 75	PPV^a^: 70% (95% CI: 66.3, 73.5)
False negative: 2	True negative: 98	NPV^b^: 98% (95% CI: 92.5, 99.5)
Sn^c^: 99% (95% CI: 96.0, 99.9)	Sp^d^: 57% (95% CI: 48.9, 64.2)	n = 350 patient admissions

^a^PPV, positive predictive value.

^b^NPV, negative predictive value.

^c^Sn, sensitivity.

^d^Sp, specificity.

#### Potential confounders

Manual validation for lab values, EKG data (QT-interval corrected by Bazett’s formula and cardiac rhythm), and telemetry order and indication showed ≥95% agreement. Manual validation of the presence of relevant comorbidities (diabetes, hypertension, and heart failure) showed 100%, 100%, and 96% agreement, respectively. However, computational validation of these comorbidities against CIHI-DAD showed 56%, 84%, and 69% agreement due to Epic missing the diagnoses in some patients with these comorbidities (Table 5). Interacting drugs were manually validated for select metabolic inhibitors (ketoconazole, clarithromycin, and bupropion), a metabolic inducer (phenytoin), and diuretics (furosemide, hydrochlorothiazide, indapamide, and metolazone) for up to 25 charts each for all administrations which showed 100%, 100%, and 93% (95% CI: 90.5, 95.7) agreement, respectively. Certain strengths of furosemide were missed in both generic name and ATC code extractions, leading to lower agreement than other interacting drugs. For electronic decision support alerts, 850,672 medication-related alerts were shown to providers in 2023. The override rate of these alerts was 98%. Pharmacists saw the largest proportion of these alerts, followed by medical residents and attending physicians. Manual validation of the override history of 65 overridden medication alerts showed 88% (95% CI: 77.2, 94.5) agreement due to the absence of some alerts from the chart. Overall, potential confounders had excellent validity (>95%) for most items but required CIHI-DAD linkage for chronic comorbidities due to false negatives in Epic.

**TABLE 5:** Diagnostic accuracy measures for comorbidities.

Diabetes			Hypertension			Heart failure		
True positive: 314	False positive: 23	PPV^a^: 93% (95% CI: 90.1, 95.4)	True positive: 421	False positive: 643	PPV: 40% (95% CI: 38.0, 41.2)	True positive: 116	False positive: 207	PPV: 36% (95% CI: 32.3, 39.7)
False negative: 246	True negative: 1,253	NPV^b^: 84% (95% CI: 82.3, 84.8)	False negative: 81	True negative: 691	NPV: 90% (95% CI: 87.4, 91.3)	False negative: 51	True negative: 1,462	NPV: 97% (95% CI: 95.8, 97.3)
Sn^c^: 56% (95% CI: 51.9, 60.2)	Sp^d^: 98% (95% CI: 97.3, 98.9)	n = 1,836 patients	Sn: 84% (95% CI: 80.4, 87.0)	Sp: 52% (95% CI: 49.1, 54.6)	n = 1,836 patients	Sn: 69% (95% CI: 61.9, 76.3)	Sp: 88% (95% CI: 85.9, 89.1)	n = 1,836 patients

^a^PPV, positive predictive value.

^b^NPV, negative predictive value.

^c^Sn, sensitivity.

^d^Sp, specificity.

#### Timestamping

The date of admission was computationally validated against CIHI-DAD for 10,065 admissions, which showed 99% agreement. Manual validation for patients for date of admission, medication administrations, lab values, EKGs, telemetry order, and medication alerts showed 100% agreement. Manual validation of telemetry discontinuation time for 84 orders showed 87% agreement (95% CI: 77.8, 93.3). Manual validation for the date of MACE (other than death) based on 25 patients showed only 36% agreement (95% CI: 18.0, 57.5), as many diagnoses were not added to the problem list or medical history until days after the event or were already present from a previous event. Unfortunately, CIHI-DAD does not abstract the precise date of diagnosis. Manual validation for the date of death for 200 patients showed 98% agreement (95% CI: 94.3, 99.2) as the date of death was off by 1 day for 5 patients. Overall, timestamping had excellent validity (>95%) for most items except nonfatal MACE outcomes and telemetry discontinuation.

## Discussion

### Principal findings

We believe that our study is the first to present a thorough and comprehensive validation of key EMR data fields for pharmacoepidemiology research using the ubiquitous Epic EMR. ERD development to provide a clear roadmap to understand which data tables and fields were required was time consuming given the vast Epic Clarity database. The iterative process required for the ERD and data validation took approximately 11 months for both the senior research data analyst and lead pharmacist author. In the end, we showed that 34 of 46 data concepts required for pharmacoepidemiologic research had excellent validity with agreement over 95%. Specifically, the demographics, exposures, most potential confounders, and most timestamping items had very high levels of agreement (>95%), as these items reside primarily in structured data fields. Exposures and their timing with very high levels of agreement are important for causal analysis with outcomes so that the exposure occurred before the outcome. However, more work is required to improve diagnoses and timing for both outcomes and comorbidities.

These problematic diagnosis data extractions for our primary outcome of MACE are important data validity issues and are due to Epic not coding acute and chronic diagnoses in a structured data field from clinical notes unless they are manually added by providers in the problem list or medical history. Even if manually added by a provider, high rates of false-positive diagnoses may occur because of timing as these outcomes may remain on the problem list or medical history even years later, and clinicians may not update the timing of a diagnosis already in a problem list if the event recurred. This led to poor agreement for ventricular arrhythmia, which can be confirmed with EKGs and telemetry (Whitaker et al., 2023). However, our data had many historical diagnoses that were not updated for the hospital admission of interest. Linking EMR data to CIHI-DAD improved the validity of diagnoses, including the phase of care. However, we join others in advocating for improvements in CIHI’s handling of diagnoses. For some comorbidities, such as hypertension and diabetes, diagnoses are mandatory to be coded in CIHI-DAD, as well as any diagnosis that lengthens hospital stay, needs additional treatment compared with maintenance, or remarkably affects the hospital treatment (Canadian Institute for Health Information, 2016; Canadian Institute for Health Information, 2022). This can lead to false negatives for some comorbidities, such as heart failure, which may not always be coded.

Natural language processing (NLP) using machine learning, by parsing clinician notes, could be helpful in finding diagnoses and making calculated assumptions about timing (Sheikhalishahi et al., 2019). For example, studies are promising for identifying pneumonia as a diagnosis and classification of heart failure severity, with PPVs and sensitivities ranging from approximately 50% to 100% (Chapman et al., 2022; Adejumo et al., 2024). However, further work may be required for NLP to provide timestamping as the onset of psychosis symptoms in another study was correct only 71% of the time (Viani et al., 2021). Future work for this study will incorporate NLP of clinician notes for identification and timing of diagnostic outcomes, which could work well for those containing specific terms such as TdP.

### Limitations of the study

Some limitations of this study include the nature of the comparator data set used, relatively small manual validation numbers for some items, and using percent agreement when there could be multiple areas of missing data. Given the >20,000 tables in Epic Clarity, it is possible, but unlikely, that there are other relevant tables that we missed or have not been activated that might validate better than the ones we used for this work. However, we iteratively validated the data to try to find the most appropriate tables for pharmacoepidemiologic research. Manual validation was conducted using a modest number of charts for each data concept because it is quite labor intensive. Validating more charts or different chart selection methods might change the level of agreement for each item. Using agreement as a validation statistic helped identify whether data items found in Epic were true, where CIHI-DAD was helpful in identifying missing data from Epic, but sometimes CIHI-DAD did not collect the required data.

### Meaning and generalizability of the study

EMR research has been growing in popularity due to the efficiency of using large numbers of real-time observational data for many important clinical and health service questions where randomized controlled trials cannot be conducted or would not be appropriate (Nordo et al., 2019; Fernainy et al., 2024). There are many different potential sources of error in EMR data that necessitate data validation to identify and correct errors (Wang et al., 2023; Verma et al., 2021; Bayley et al., 2013). Although this study was only conducted at a single site using Epic, the results and example ERD show the types of data that could be relied on based on our specific extractions from Epic Clarity versus data that require additional work to ensure validity, such as diagnoses. However, EMR data from any institution should be validated before conducting research to check the validity of each key data field.

Epic is a rapidly growing EMR that is already dominant in North America (Becker’s Hospital Review, 2024; Lougheed, 2019). Sharing detailed ERDs for Epic among researchers and institutions could help reduce major inefficiencies when setting up an EMR data set for research. The ERD we created is more conceptual than the OMOP ERD, which is a standard for EMR data extraction (Observational Health Data Sciences and Informatics, 2025). However, it shows the types of data and linkages in Epic Clarity that we validated.

Epic also has an anonymized data set known as COSMOS from >300 million patients and 1,800 hospitals worldwide (Epic Systems, 2025b). Institutions can opt-in to join the COSMOS data set, which contains demographics, hospital admission details, diagnoses, medications, and more (Tarabichi et al., 2021). However, free text in clinical notes is not submitted to COSMOS, which could miss diagnoses not documented in structured data fields (Tarabichi et al., 2021). Converting EMR data to a common data model, such as OMOP or the developing logical data model from CIHI, would lead to a standard structure of data allowing for collaborative research with institutions using Epic and other EMRs (Observational Health Data Sciences and Informatics, 2025; Canadian Institute for Health Information, 2024).

## Conclusion

Once an accurate ERD was formulated, most required data concepts for pharmacoepidemiologic research, including patient demographics and admission information, exposures, most potential confounders such as interacting drugs and lab results, and timestamping, were validated well. However, some diagnoses require a linked data source or the development of advanced techniques, such as NLP, to improve validity.

## Data Availability

The raw data supporting the conclusions of this article will be made available by the authors, without undue reservation.
