# Supplementary material for: Characterization and validation of electronic medical record data for pharmacoepidemiologic research
Source: Front Pharmacol. 2026 Jul 21;17:1812502. doi: 10.3389/fphar.2026.1812502 (PMC13433748; doi:10.3389/fphar.2026.1812502)
Supplement: Supplementary file 1 [file Table1.docx]

**Table A.1: STARE-HI Checklist**

| **STARE-HI item** | **Item detailed in manuscript? (√/-)** |
| --- | --- |
| **Title** | √ |
| **Abstract** | √ |
| **Keywords** | √ |
| **Introduction** | √ |
| Scientific background | √ |
| Rationale for the study | √ |
| Objectives of the study | √ |
| **Study context** | √ |
| Organizational setting | √ |
| System details and system in use | √ |
| **Methods** | √ |
| Study design | √ |
| Theoretical background | √ |
| Participants | √ |
| Study flow | √ |
| Outcome measures or evaluation criteria | √ |
| Methods for data acquisition and measurement | √ |
| Methods for data analysis | √ |
| **Results** | √ |
| Demographic and other study coverage data | √ |
| Unexpected events during the study | √ |
| Study findings and outcome data | √ |
| Unexpected observations | √ |
| **Discussion** | √ |
| Answers to study questions | √ |
| Strengths and weaknesses of the study | √ |
| Results in relation to other studies | √ |
| Meaning and generalizability of the study | √ |
| Unanswered and new questions | √ |
| **Conclusion** | √ |
| **Authors’ contribution** | √ |
| **Competing interests** | √ |
| **Acknowledgement** | √ |
