# Supplementary material for: Characterization and validation of electronic medical record data for pharmacoepidemiologic research
Source: Front Pharmacol. 2026 Jul 21;17:1812502. doi: 10.3389/fphar.2026.1812502 (PMC13433748; doi:10.3389/fphar.2026.1812502)
Supplement: Supplementary file 2 [file Table2.docx]

**Table A.2: Diagnostic Codes Used for Outcomes**

| **MACE^a^ Outcome** | **ICD-10^b^ Code** |
| --- | --- |
| Alcohol withdrawal seizure | F10.3 |
| Seizure due to alcohol withdrawal | F10.3 |
| Drug withdrawal seizure | F19.399, R56.88 |
| Withdrawal seizures | F19.399, R56.88 |
| Conversion disorder (functional neurological symptom disorder), with attacks or seizures | F44.5 |
| Conversion disorder with seizures or convulsions | F44.5 |
| Functional neurological symptom disorder with attacks or seizures | F44.5 |
| Pseudoseizure | F44.5 |
| Pseudoseizures | F44.5 |
| Psychiatric pseudoseizure | F44.5 |
| Psychogenic nonepileptic seizure | F44.5 |
| Psychogenic syncope | F48.8 |
| Syncope, psychogenic | F48.8 |
| Epilepsy with simple partial seizures | G40.10 |
| Focal motor seizure | G40.10 |
| Partial seizure disorder | G40.10 |
| Seizure disorder, focal motor | G40.10 |
| Complex partial seizure | G40.20 |
| Complex partial seizure disorder | G40.20 |
| Complex partial seizures | G40.20 |
| Epilepsy with partial complex seizures | G40.20 |
| Seizure disorder, complex partial | G40.20 |
| Temporal lobe seizure | G40.20 |
| Absence seizure | G40.30 |
| Epilepsy seizure, generalized, convulsive | G40.30 |
| Epileptic seizure, generalized, convulsive | G40.30 |
| Myoclonic seizures | G40.30 |
| Seizure, absence | G40.30 |
| Seizure disorder, generalized convulsive, intractable | G40.31 |
| Lennox-Gastaut syndrome with tonic seizures | G40.40 |
| Generalized tonic-clonic seizure | G40.60 |
| Grand mal seizure | G40.60 |
| Grand mal seizures, unspecified (with or without petit mal), not stated as intractable | G40.60 |
| Seizure disorder, grand mal | G40.60 |
| Seizure grand mal | G40.60 |
| Seizure, grand mal | G40.60 |
| Tonic clonic seizures | G40.60 |
| Tonic-clonic seizures | G40.60 |
| Abdominal seizure | G40.80 |
| Recurrent seizures | G40.90 |
| Non-convulsive seizure disorder with status epilepticus | G41.8 |
| Carotid sinus syncope | G90.0 |
| Cardiac arrest with successful resuscitation | I46.0 |
| Sudden cardiac death | I46.1 |
| Asystole | I46.9 |
| Cardiac arrest | I46.9 |
| Cardiac arrest during or resulting from a procedure | I46.9 |
| Cardiac arrest with pulseless electrical activity | I46.9 |
| Cardiac arrest, unspecified | I46.9 |
| Cardiac asystole | I46.9 |
| Cardiopulmonary arrest | I46.9 |
| Nontraumatic cardiac arrest | I46.9 |
| Sudden cardiac arrest | I46.9 |
| Traumatic cardiac arrest | I46.9 |
| Paroxysmal supraventricular tachycardia | I47.1 |
| Paroxysmal SVT (supraventricular tachycardia) | I47.1 |
| PSVT (paroxysmal supraventricular tachycardia) | I47.1 |
| Supraventricular tachycardia | I47.1 |
| Supraventricular tachycardia, nonsustained | I47.1 |
| SVT (supraventricular tachycardia) | I47.1 |
| Monomorphic ventricular tachycardia | I47.2 |
| Nonsustained ventricular tachycardia | I47.2 |
| Non-sustained ventricular tachycardia | I47.2 |
| NSVT (nonsustained ventricular tachycardia) | I47.2 |
| Paroxysmal ventricular tachycardia | I47.2 |
| Polymorphic ventricular tachycardia | I47.2 |
| Re-entry ventricular tachycardia | I47.2 |
| Right ventricular outflow tract ventricular tachycardia | I47.2 |
| Torsades de pointes | I47.2 |
| V tach | I47.2 |
| Ventricular tachyarrhythmia | I47.2 |
| Ventricular tachycardia | I47.2 |
| Ventricular tachycardia (paroxysmal) | I47.2 |
| Ventricular tachycardia with normal heart | I47.2 |
| Ventricular tachycardia, incessant | I47.2 |
| Ventricular tachycardia, monomorphic | I47.2 |
| Ventricular tachycardia, non-sustained | I47.2 |
| Ventricular tachycardia, polymorphic | I47.2 |
| Ventricular tachycardia, sustained | I47.2 |
| VT (ventricular tachycardia) | I47.2 |
| V-tach | I47.2 |
| Wide QRS ventricular tachycardia | I47.2 |
| Paroxysmal tachycardia | I47.9 |
| Paroxysmal tachycardia, unspecified | I47.9 |
| Tachycardia, paroxysmal | I47.9 |
| Ventricular fibrillation | I49.00 |
| VF (ventricular fibrillation) | I49.00 |
| Arrhythmia, atrial | I49.8 |
| Arrhythmia, AV node | I49.8 |
| Arrhythmia, ventricular | I49.8 |
| Atrial arrhythmia | I49.8 |
| Atrial dysrhythmia | I49.8 |
| AV node arrhythmia | I49.8 |
| Bigeminy | I49.8 |
| Bradyarrhythmia | I49.8 |
| Brugada syndrome | I49.8 |
| Cardiac fibrillation | I49.8 |
| Fluttering heart | I49.8 |
| Junctional rhythm | I49.8 |
| Long QT syndrome | I49.8 |
| Long Q-T syndrome | I49.8 |
| Other specified cardiac arrhythmias | I49.8 |
| Prolonged QT interval syndrome | I49.8 |
| Prolonged QT syndrome | I49.8 |
| Sinus arrhythmia | I49.8 |
| Supraventricular arrhythmia | I49.8 |
| Tachyarrhythmia | I49.8 |
| Ventricular arrhythmia | I49.8 |
| Ventricular bigeminy | I49.8 |
| Ventricular dysrhythmia | I49.8 |
| Ventricular trigeminy | I49.8 |
| Wandering atrial pacemaker | I49.8 |
| Abnormal heart rhythm | I49.9 |
| Arrhythmia | I49.9 |
| Arrhythmia requiring replacement of cardiac pacemaker | I49.9 |
| Cardiac arrhythmia | I49.9 |
| Cardiac arrhythmia, unspecified | I49.9 |
| Cardiac dysrhythmia | I49.9 |
| Dysrhythmia | I49.9 |
| Dysrhythmia, cardiac | I49.9 |
| Dysrhythmias | I49.9 |
| Irregular cardiac rhythm | I49.9 |
| Irregular heart beat | I49.9 |
| Irregular heart beats | I49.9 |
| Irregular heart rate | I49.9 |
| Irregular heart rhythm | I49.9 |
| Irregular heartbeat | I49.9 |
| Seizure cerebral | I64 |
| Orthostatic syncope | I95.1 |
| Syncope due to orthostatic hypotension | I95.1 |
| Seizure disorder during pregnancy, delivered | O99.801, R56.80 |
| Seizure disorder in pregnancy | O99.803, R56.88 |
| Cough syncope syndrome | R05 |
| Syncope, tussive | R05 |
| Tussive syncope | R05 |
| Near syncope | R42 |
| Postural dizziness with near syncope | R42 |
| Postural dizziness with presyncope | R42 |
| Pre-syncope | R42 |
| Syncope, near | R42 |
| Atypical syncope | R55 |
| Black-out (not amnesia) | R55 |
| Blackout spell | R55 |
| Cardiac related syncope | R55 |
| Cardiac syncope | R55 |
| Convulsive syncope | R55 |
| Drop attack | R55 |
| Effort syncope | R55 |
| Episode of loss of consciousness | R55 |
| Episode of syncope | R55 |
| Faint | R55 |
| Fainting | R55 |
| Fainting episodes | R55 |
| Fainting spell | R55 |
| Faintness | R55 |
| Micturition syncope | R55 |
| Neurocardiogenic syncope | R55 |
| Postural syncope | R55 |
| Syncopal episodes | R55 |
| Syncope | R55 |
| Syncope and collapse | R55 |
| Syncope, cardiogenic | R55 |
| Syncope, effort | R55 |
| Syncope, non cardiac | R55 |
| Syncope, vasovagal | R55 |
| Transient LOC (loss of consciousness) | R55 |
| Transient loss of consciousness | R55 |
| Vaso vagal episode | R55 |
| Vasodepressor syncope | R55 |
| Vasovagal attack | R55 |
| Vasovagal episode | R55 |
| Vasovagal near syncope | R55 |
| Vasovagal near-syncope | R55 |
| Vasovagal reaction | R55 |
| Vaso-vagal reaction | R55 |
| Vasovagal response | R55 |
| Vasovagal syncope | R55 |
| Vasovagal syncopes | R55 |
| Vasovagal syndrome | R55 |
| Febrile seizure | R56.09 |
| Febrile seizures | R56.09 |
| Generalized seizure disorder | R56.80 |
| Secondary seizure disorder | R56.80 |
| Seizure disorder | R56.80 |
| Seizure disorder, secondary | R56.80 |
| Seizure disorder, so described | R56.80 |
| First time seizure | R56.88 |
| Focal seizure | R56.88 |
| Focal seizures | R56.88 |
| Generalized convulsive seizure | R56.88 |
| Generalized seizures | R56.88 |
| Motor seizure | R56.88 |
| New onset seizure | R56.88 |
| Nocturnal seizures | R56.88 |
| Partial seizure | R56.88 |
| Partial seizures | R56.88 |
| Post traumatic seizure | R56.88 |
| Post traumatic seizure disorder | R56.88 |
| Provoked seizure | R56.88 |
| Provoked seizures | R56.88 |
| Seizure | R56.88 |
| Seizure in child | R56.88 |
| Seizure in childhood | R56.88 |
| Seizure in response to acute event | R56.88 |
| Seizure with provoking factor | R56.88 |
| Seizure-like activity | R56.88 |
| Seizures | R56.88 |
| Seizures, generalized convulsive | R56.88 |
| Witnessed seizure | R56.88 |
| Seizures due to metabolic disorder | R56.88, E88.9 |
| Alcohol related seizure | R56.88, Y15 |
| Drug-induced seizure | R56.88, Y57.9 |
| Death | R99 |
| Death due to natural causes | R99 |
| Death in hospital | R99 |
| Deceased | R99 |
| DOA (dead on arrival) | R99 |
| H/O absence seizures | Z86.6 |
| History of absence seizures | Z86.6 |
| History of petit-mal seizures | Z86.6 |
| History of seizure disorder | Z86.6 |
| Hx of seizure disorder | Z86.6 |
| H/O cardiac arrest | Z86.78 |
| H/O paroxysmal supraventricular tachycardia | Z86.78 |
| H/O supraventricular tachycardia | Z86.78 |
| H/O sustained ventricular fibrillation | Z86.78 |
| History of cardiac arrest | Z86.78 |
| History of paroxysmal supraventricular tachycardia | Z86.78 |
| History of sudden cardiac arrest | Z86.78 |
| History of sudden cardiac arrest successfully resuscitated | Z86.78 |
| History of ventricular tachycardia | Z86.78 |
| Hx of cardiac arrest | Z86.78 |
| Personal history of supraventricular tachycardia | Z86.78 |
| H/O syncope | Z87.8 |
| History of idiopathic seizure | Z87.8 |
| History of seizure | Z87.8 |
| History of seizures | Z87.8 |
| History of syncope | Z87.8 |
| Hx of idiopathic seizure | Z87.8 |
| Hx of syncope | Z87.8 |

*^a^ MACE = major adverse cardiac event; ^b^ ICD-10 = International Classification of Diseases, Tenth Revision*
