# Supplementary material for: Characterization and validation of electronic medical record data for pharmacoepidemiologic research
Source: Front Pharmacol. 2026 Jul 21;17:1812502. doi: 10.3389/fphar.2026.1812502 (PMC13433748; doi:10.3389/fphar.2026.1812502)
Supplement: Supplementary file 3 [file Table3.docx]

**Table A.3: Search Strings Used in the Epic Electronic Medical Record for Manual Chart Review**

| Item of interest | Search string |
| --- | --- |
| Hypertension | Hypertension OR htn OR high blood pressure |
| Diabetes | Diabetes |
| Heart Failure | Heart failure OR CHF OR HFREF OR HFMEF OR HFMREF OR HFPEF OR HFIMPEF |
| Lab values | (STJ OR HRLMP OR ML) AND (potassium OR magnesium OR calcium OR troponin) |
